# Supplementary material for: Optimizing Sparse Testing for Genomic Prediction of Plant Breeding Crops
Source: Genes (Basel). 2023 Apr 17;14(4):927. doi: 10.3390/genes14040927 (PMC10137724; doi:10.3390/genes14040927)
Supplement: Supplementary file 1 [file genes-14-00927-s001.zip › Supplementary Material - Result Small Data Sets.pdf]

Maize_250 small data set

In the smaller version of the previous data set (with only 250 lines) we observe that in terms of APC, in all scenarios of testing proportions, the best prediction performance was recorded under a multi-trait framework and the worst under a uni-trait framework (**Figure S1, Table S1**). However, the superiority of the multi-trait model over the uni-trait model was more modest in the whole maize data set, in which a larger superiority was detected. Nevertheless, we also observed a similar prediction performance between the five percentage of testing even in the scenario with 85% (0.85) of testing. No significant differences were observed between the four sparse testing methods, as we see only slight differences in the five different percentage of testing. For example, under the 15% (0.15) of testing set in the multi-trait model, M3 outperformed M1, M2 and M4 by 15.4, 2.9 and 6.3% respectively, while under the uni-trait model, M3 only outperformed M1, M2 and M4 by 1.0, 0.7 and 7.7% respectively. Under the 85% (0.85) testing multi-trait method, M4 outperformed methods M1, M2 and M3 by 10.67%, 10.19% and 6% respectively. No relevant differences were observed between methods in most of the proportion of testing evaluated.

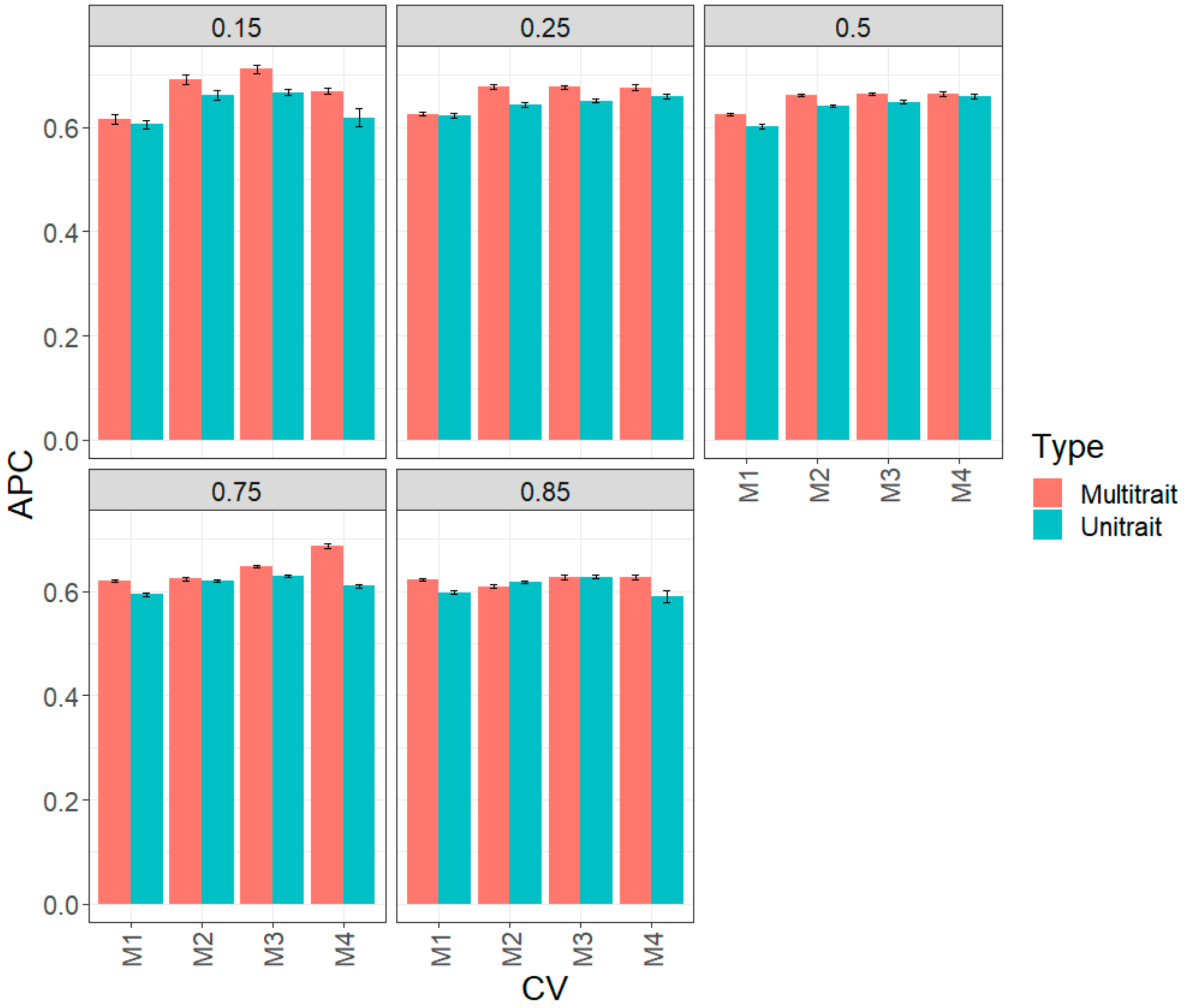

Figure S1. Prediction performance for small maize_250 data set in terms of average Pearson´s correlation (APC) of the four methods of sparse testing (M1, M2, M3 and M4) under unit-trait and multi-trait models for 5 percentage of testing 15% (0.1), 25% (0.25), 50% (0.5), 75% (0.75) and 85% (0.85).

The multi-trait model was better than the uni-trait model in terms of NRMSE (**Figure S2, Table S1**), but the superiority was lower than under the maize data set. The best predictions in terms of NRMSE were under methods M3 and M4. Under the percentage of testing of 25% (0.25), we observed that in the uni-trait model, M4 and uni-trait outperformed methods M1, M2 and M3 by 8.2, 11.9 and 3.2 % respectively, but under the multi-trait model M4 outperformed methods M1, M2 and M3 by 6.8, 8.6 and 0.3 % respectively. While under the percentage of testing of 50% (0.5) and under the uni-trait model, the method M4 outperformed methods M1, M2 and M3 by 9.1, 15.6

and 4.7 % respectively. Nevertheless, for the other percentages of testing, no relevant differences were observed between methods.

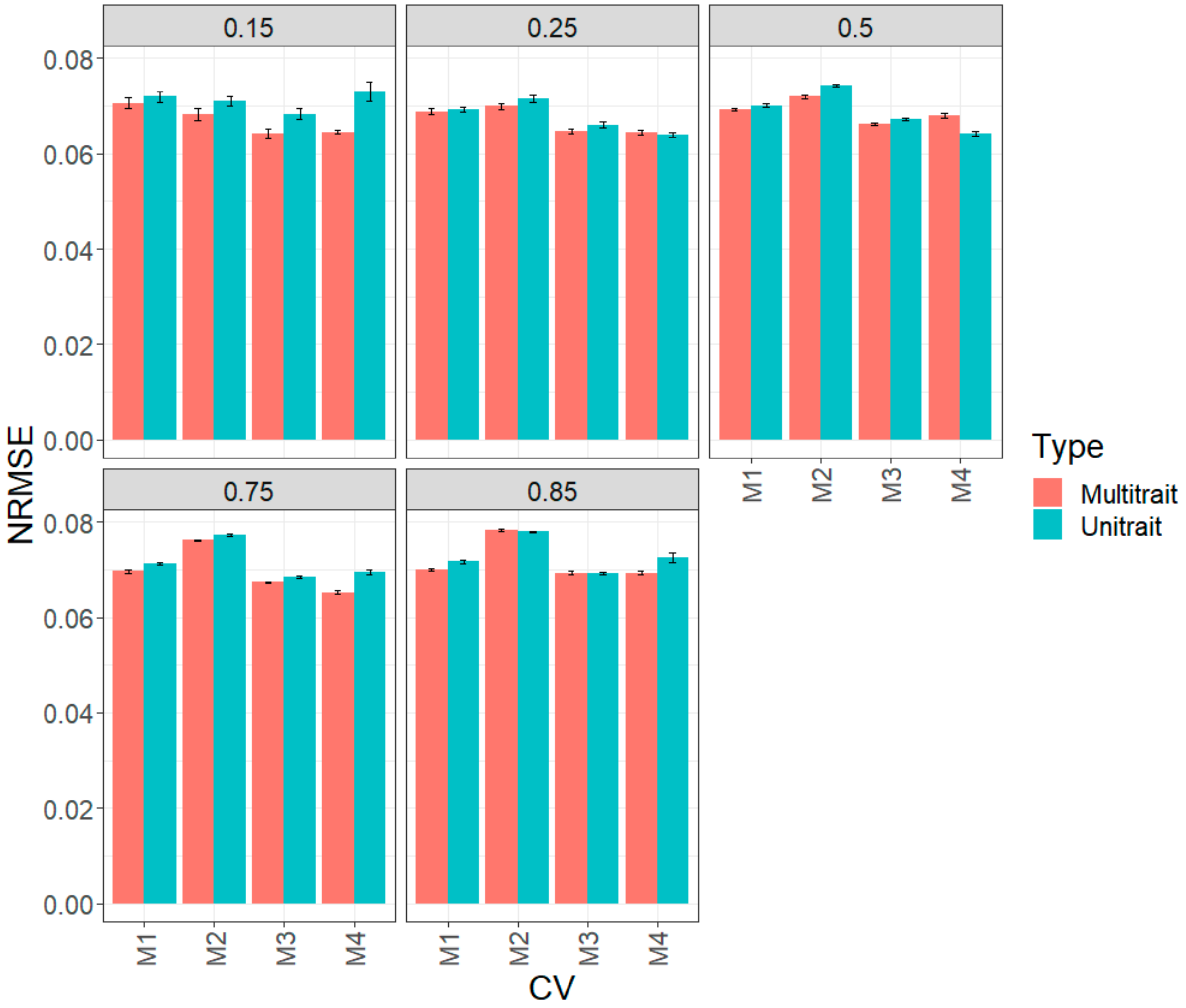

Figure S2. Prediction performance for small maize_250 data set in terms of normalized root mean square error (NRMSE) of the four methods of sparse testing (M1, M2, M3 and M4) under unit-trait and multi-trait models for 5 percentage of testing 15% (0.1), 25% (0.25), 50% (0.5), 75% (0.75) and 85% (0.85).

Table S1. Prediction performance for the small maize_250 data in terms of normalized root mean square error (NRMSE) and Average Pearson´s correlation (APC) of the four sparse testing methods (CV) under the following proportion of testing (Prop_testing): 15% (0.15), 25% (0.2), 50% (0.5), 75% (0.75) and 85% (0.85). NRMSE_SE denotes the standard error of NRMSE and APC_SE denotes the standard error of APC.

Dataset	CV	Prop_testing	Type	NRMSE	NRMSE_SE	APC	APC_SE
Maize_250	M1	0.15	Multitrait	0.071	0.002	0.616	0.019
Maize_250	M1	0.15	Unitrait	0.072	0.002	0.606	0.017
Maize_250	M1	0.25	Multitrait	0.069	0.001	0.626	0.009
Maize_250	M1	0.25	Unitrait	0.069	0.001	0.623	0.009
Maize_250	M1	0.5	Multitrait	0.069	0.001	0.625	0.006
Maize_250	M1	0.5	Unitrait	0.070	0.001	0.602	0.009
Maize_250	M1	0.75	Multitrait	0.070	0.001	0.621	0.005
Maize_250	M1	0.75	Unitrait	0.071	0.001	0.594	0.007
Maize_250	M1	0.85	Multitrait	0.070	0.001	0.622	0.005
Maize_250	M1	0.85	Unitrait	0.072	0.001	0.598	0.006
Maize_250	M2	0.15	Multitrait	0.068	0.003	0.691	0.017
Maize_250	M2	0.15	Unitrait	0.071	0.002	0.662	0.018
Maize_250	M2	0.25	Multitrait	0.070	0.001	0.677	0.009
Maize_250	M2	0.25	Unitrait	0.072	0.002	0.643	0.009
Maize_250	M2	0.5	Multitrait	0.072	0.001	0.661	0.005
Maize_250	M2	0.5	Unitrait	0.074	0.001	0.641	0.005
Maize_250	M2	0.75	Multitrait	0.076	0.000	0.624	0.005
Maize_250	M2	0.75	Unitrait	0.077	0.000	0.620	0.004
Maize_250	M2	0.85	Multitrait	0.078	0.000	0.609	0.006
Maize_250	M2	0.85	Unitrait	0.078	0.000	0.618	0.005
Maize_250	M3	0.15	Multitrait	0.064	0.002	0.712	0.016

Maize_250	M3	0.15	Unitrait	0.068	0.002	0.667	0.012
Maize_250	M3	0.25	Multitrait	0.065	0.001	0.677	0.008
Maize_250	M3	0.25	Unitrait	0.066	0.001	0.651	0.007
Maize_250	M3	0.5	Multitrait	0.066	0.001	0.664	0.007
Maize_250	M3	0.5	Unitrait	0.067	0.001	0.649	0.005
Maize_250	M3	0.75	Multitrait	0.067	0.000	0.648	0.004
Maize_250	M3	0.75	Unitrait	0.069	0.001	0.629	0.005
Maize_250	M3	0.85	Multitrait	0.069	0.001	0.627	0.008
Maize_250	M3	0.85	Unitrait	0.069	0.001	0.628	0.005
Maize_250	M4	0.15	Multitrait	0.065	0.001	0.669	0.011
Maize_250	M4	0.15	Unitrait	0.073	0.004	0.619	0.035
Maize_250	M4	0.25	Multitrait	0.064	0.001	0.677	0.010
Maize_250	M4	0.25	Unitrait	0.064	0.001	0.660	0.008
Maize_250	M4	0.5	Multitrait	0.068	0.001	0.664	0.009
Maize_250	M4	0.5	Unitrait	0.064	0.001	0.660	0.009
Maize_250	M4	0.75	Multitrait	0.065	0.001	0.687	0.008
Maize_250	M4	0.75	Unitrait	0.070	0.001	0.610	0.007
Maize_250	M4	0.85	Multitrait	0.069	0.001	0.627	0.008
Maize_250	M4	0.85	Unitrait	0.073	0.002	0.590	0.022

Wheat_250 small data set

In the smaller version of the wheat data set (with 250 lines) in terms of APC, we did not observe any significant differences between the multi-trait and uni-trait models in all scenarios of testing proportions (**Figure S3, Table S2**). Nevertheless, we also observed a similar prediction performance between the five percentage of testing even in the scenario with 85% (0.85) of testing, that is, the prediction performance does not decrease as the percentage of testing increases. We

observe in **Figure S3** only slight differences between the four sparse testing methods. For example, under the 15% (0.15) of testing set the M3 in the multi-trait model, outperformed M1, M2 and M4 by 5.0, 1.0 and 2.0% respectively, while under the uni-trait model M3 only outperformed M1, M2 and M4 by 7.4, 2.6 and 1.1% respectively. Under the 25% (0.25) testing and multi-trait method, M3 outperformed methods M1, M2 and M4 by 4.8%, 1.2% and 0.005% respectively. No relevant differences were observed between methods in most of the proportion of testing evaluated.

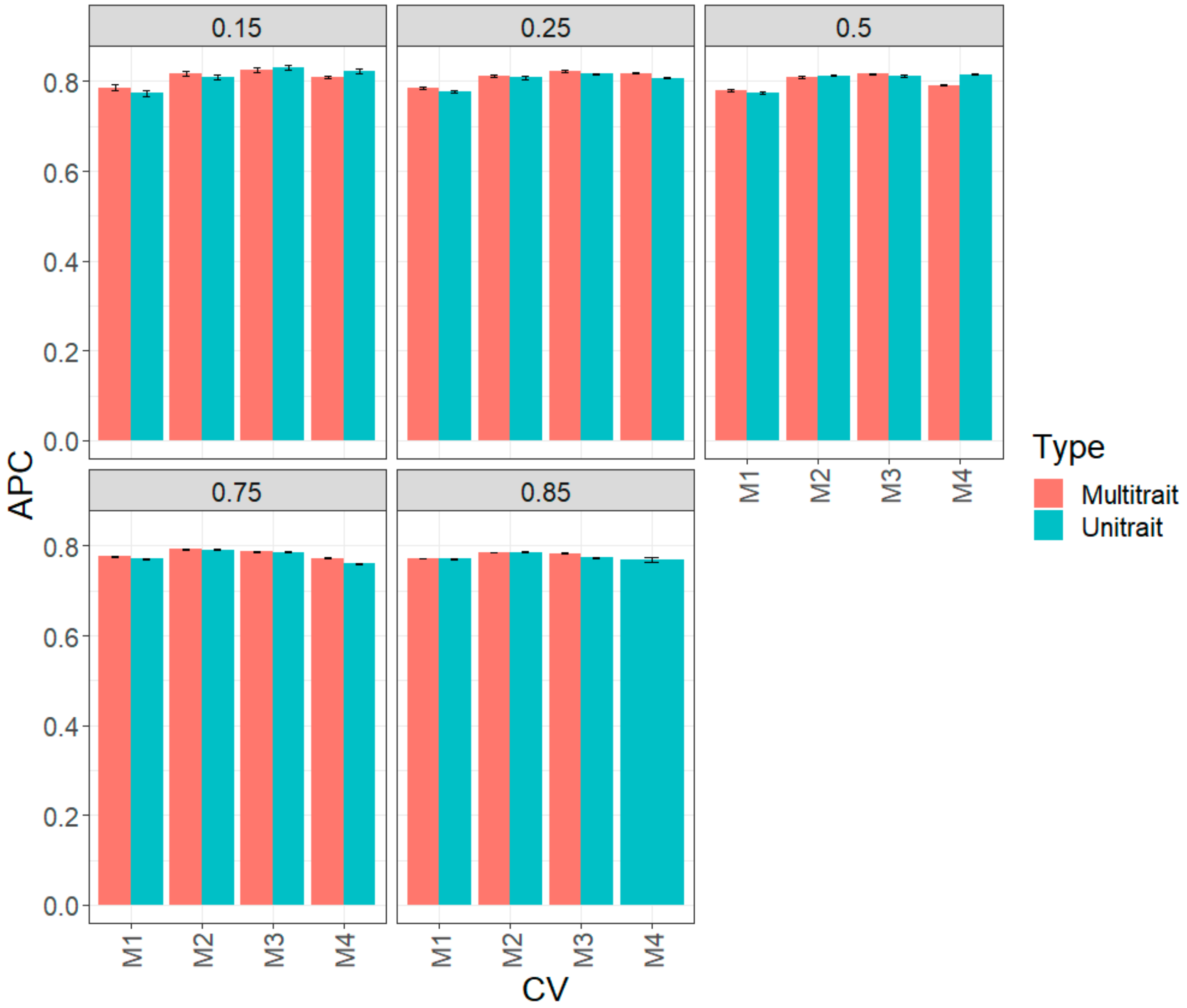

Figure S3. Prediction performance for wheat_250 small data set in terms of average Pearson´s correlation (APC) of the four methods of sparse testing (M1, M2, M3 and M4) under unit-trait and multi-trait models for 5 percentage of testing 15% (0.1), 25% (0.25), 50% (0.5), 75% (0.75) and 85% (0.85).

Now, the multi-trait model was only slightly better than the uni-trait model in terms of NRMSE (**Figure S4, Table S2**). Likewise, the best predictions in terms of NRMSE were under method M3. Under the percentage of testing of 15% (0.15) we observed that method M4 in the uni-trait model outperformed methods M1 and M2 by 7.4 and 2.72 % respectively, but under the multi-trait model, M4 outperformed only M1 by 3.2%. Under the percentage of testing of 25% (0.25) the method M3 under multi-trait model outperformed methods M1, M2 and M3 by 9.5, 3.8 and 3.9 % respectively. While under the uni-trait model, M3 outperformed M1, M2 and M3 by 7.4, 3.7 and 5.1 % respectively. Nevertheless, for the other percentage of testing, no relevant differences were observed between methods (**Figure S4, Table S2**).

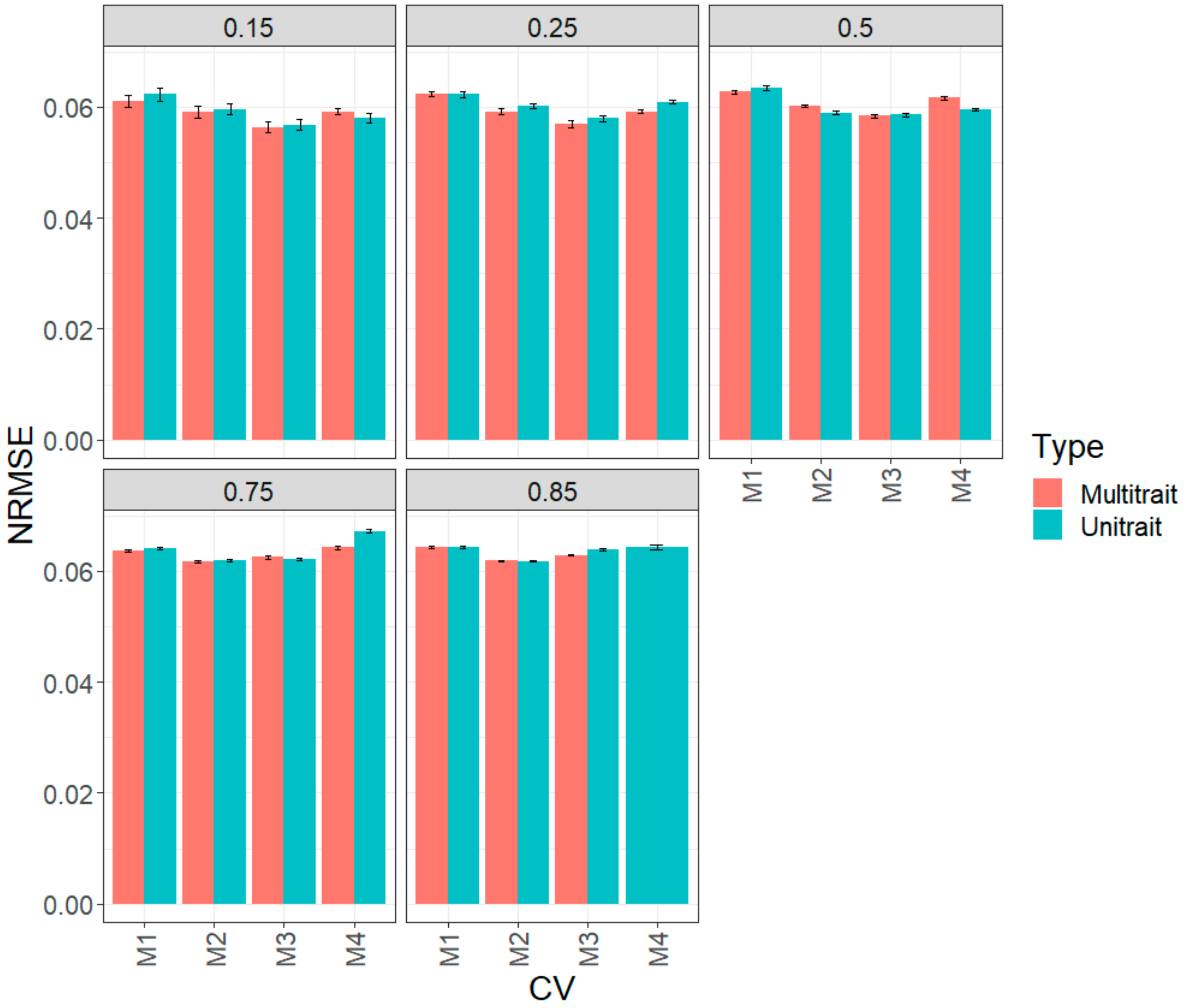

Figure S4. Prediction performance for wheat_250 small data set in terms of normalized root mean square error (NRMSE) of the four methods of sparse testing (M1, M2, M3 and M4) under unit-trait and multi-trait models for 5 percentage of testing 15% (0.1), 25% (0.25), 50% (0.5), 75% (0.75) and 85% (0.85).

Table S2. Prediction performance for the wheat_250 small data in terms of normalized root mean square error (NRMSE) and Average Pearson´s correlation (APC) of the four sparse testing methods (CV) under the following proportion of testing (Prop_testing): 15% (0.15), 25% (0.2), 50% (0.5), 75% (0.75) and 85% (0.85). NRMSE_SE denotes the standard error of NRMSE and APC_SE denotes the standard error of APC

Dataset	CV	Prop_testing	Type	NRMSE	NRMSE_SE	APC	APC_SE
Wheat_250	M1	0.15	Multitrait	0.061	0.002	0.786	0.014
Wheat_250	M1	0.15	Unitrait	0.062	0.002	0.774	0.014
Wheat_250	M1	0.25	Multitrait	0.062	0.001	0.785	0.007
Wheat_250	M1	0.25	Unitrait	0.062	0.001	0.777	0.008
Wheat_250	M1	0.5	Multitrait	0.063	0.001	0.781	0.004
Wheat_250	M1	0.5	Unitrait	0.063	0.001	0.775	0.004
Wheat_250	M1	0.75	Multitrait	0.064	0.001	0.776	0.003
Wheat_250	M1	0.75	Unitrait	0.064	0.000	0.771	0.002
Wheat_250	M1	0.85	Multitrait	0.064	0.001	0.772	0.002
Wheat_250	M1	0.85	Unitrait	0.064	0.000	0.771	0.002
Wheat_250	M2	0.15	Multitrait	0.059	0.002	0.818	0.012
Wheat_250	M2	0.15	Unitrait	0.060	0.002	0.810	0.012
Wheat_250	M2						
